# Aux/IAAs: specificity and redundancy

**DOI:** 10.1080/15592324.2025.2530541

**Published:** 2025-07-10

**Authors:** Qiming Wen, Qian Gong, Huaying Yu, Panyu Yang

**Affiliations:** a Key Laboratory of Ecology of Rare and Endangered Species and Environmental Protection, Ministry of Education, Guangxi Normal University, Guilin, China; b University Engineering Research Center of Bioinformation and Genetic Improvement of Specialty Crops, Guangxi, Guilin, China

**Keywords:** Aux/IAAs, loss-of-function mutants, gain-of-function mutants, redundancy, specificity

## Abstract

*Auxin/Indole-3-Acetic Acids*(*Aux/IAAs*), a class of early auxin-responsive genes, encode short-lived nuclear proteins that play pivotal roles in auxin signaling. In vascular plants, *Aux/IAA* genes form large families-such as the 29 members in Arabidopsis, exhibiting both functional redundancy and specificity. Canonical Aux/IAA proteins contain four conserved domains and mediate nuclear auxin response by interacting with Transport Inhibitor Response 1/Auxin Signaling F‐box (TIR1/AFB) auxin receptors and Auxin Response Factor (ARF) transcription factors. Loss- and gain-of-function mutants have been instrumental in dissecting the roles of individual *Aux/IAAs*. Recent studies have also uncovered the mechanism of non-canonical Aux/IAAs, which lack one or more conserved domains and regulate auxin signaling through distinct pathways. This review summarizes the structural features of Aux/IAA proteins, the functional diversity of non-canonical members, the phenotypic effects of their mutants, and their expression patterns. These findings reveal a hierarchical regulatory network of the *Aux/IAA* gene family in auxin signaling – balancing robustness through functional redundancy and precision and flexibility through member-specific functions in plant growth and development.

## 1. Introduction

Auxin, the first-identified plant hormone, regulates a wide range of plant growth and developmental processes, including hypocotyl elongation, root growth inhibition, lateral and adventitious root formation, tropic response, and apical dominance-by modulating cell division, enlargement, and differentiation.^1–4^ Auxin mediates its effects through two interconnected pathways: the canonical TIR1/AFB-dependent pathway and various non-canonical signaling mechanism. Plants utilize both non-canonical and canonical signaling pathways to coordinately control complex developmental processes.^5,6^

In recent years, research on non-canonical auxin signaling pathways has been highly active and has made significant progress.^6,7^ Non-canonical auxin signaling functions at multiple regulatory levels. For instance, AFB1 can trigger rapid intracellular auxin responses independently of transcriptional regulation.^8–10^ Although the role of ABP1 (Auxin Binding Protein 1) as an auxin receptor has been debated, it still remains a focus of auxin signaling research. ABP1 and its homologs ABLs (ABP1-Like Proteins) cooperate with transmembrane kinase TMKs (Transmembrane Kinases) to mediate auxin signaling at the plasma membrane. ABP1/ABLs perceive extracellular auxin and triggers the interaction between ABP1/ABLs and the extracellular domain of TMK，leading to TMK phosphorylation and diverse auxin response.^11,12^

The most well-characterized mechanism is the nuclear TIR1/AFB-Aux/IAA-ARF transcriptional pathway, which has been extensively studied over the past decades.^3^ In this canonical pathway, the small-molecule auxin (IAA) functions as a “molecular glue”，promoting the interaction between the TIR1/AFB receptors and Aux/IAA transcriptional repressors. This association triggers the polyubiquitination and subsequent proteasomal degradation of Aux/IAA proteins.^13–15^ Then, the removal of the Aux/IAA repressors releases their inhibition on ARF transcription factors, ultimately activating auxin-responsive genes.^15,16^

Beyond their established role in the SCF E3 ubiquitin ligase complex, recent studies reveal that TIR1/AFB proteins possess adenylate cyclase (AC) activity, catalyzing the production of the secondary messenger cAMP. Intriguingly, disruption of TIR1/AFB AC activity does not impair auxin-induced Aux/IAA degradation, but significantly attenuates transcriptional reprogramming and auxin-mediated shoot and root phenotype.^17,18^ These findings challenge the traditional model, in which Aux/IAA degradation is the central driver in auxin response, and instead suggest a dual mechanism involving both proteolytic and cAMP-dependent signaling. Notably, the AC activity of TIR1/AFB requires functional interaction with Aux/IAA proteins. In vitro assay demonstrate that neither auxin alone nor stabilized the axr2/axr3 mutant proteins can activate AC activity, whereas the combinations of auxin and wild-type AXR7/AXR17 proteins increase cAMP production,^17,18^ which suggests a previously unrecognized role for Aux/IAA proteins beyond their function as repressors.

The auxin-induced transcriptional reprogramming relies on three core components: TIR1/AFBs, Aux/IAAs, and ARFs. In most plants, each component is encoded by a multigene family, enabling the formation of a vast combinatorial network to precisely sense and respond to dynamic auxin fluctuations.^19^ For example, there are 6 *TIR1/AFB*, 29 *Aux/IAA* and 23 *ARF* genes in Arabidopsis thaliana. The *Aux/IAA* genes are among the earliest and primary auxin-responsive genes exhibiting rapid transcriptional activation of auxin treatment (typically within 1 h) that does not require de novo protein synthesis.^20^ These genes encode short-lived nuclear proteins, and numerous mutations have been isolated and characterized in Arabidopsis through genetic and biochemical studies, revealing their functional diversity.^21–23^ Notably, the *Aux/IAA* gene family is widely conserved among plants, with members identified in over 30 plant species, including dicots (e.g, arabidopsis, soybean, tomato) and monocots (e.g., rice, maize, wheat). Family size varies significantly, ranging from 1 to 119, with higher plants generally possess more *Aux/IAA* members than lower plants.^21^ This expansion suggests both functional redundancy and specialization among *Aux/IAA* members.

This review synthesizes current knowledge of *Aux/IAA* genes, examining their structural features, mutant phenotypes, and expression patterns, while highlighting recent discoveries regarding non-canonical Aux/IAA proteins. We aim to provide insights into how plants balance redundancy and specificity within auxin signaling networks.

## 2. Structural features of Aux/IAA proteins

Most Aux/IAA proteins share four conserved domains (I, II, III and IV).^24^ Domain I confer the Aux/IAA protein transcriptional repression property through an ethylene response factor (ERF)‐associated amphiphilic repression (EAR) motif “LxLxL”. This motif is able to recruit the corepressor TOPLESS (TPL) and histone deacetylase (HDACs), leading to chromatin condensation and suppression of auxin-responsive gene expression.^25^ Domain II contains a transferable degron motif “GWPPV” that mediates protein stability. Mutation in this motif significantly reduced the degradation rate of Aux/IAA proteins.^26^ This conserved sequence is responsible for the interaction with the TIR1/AFB auxin receptor complex, targeting Aux/IAA proteins for proteasomal degradation. Notably, the flanking sequence adjacent to the degron also play a crucial role in regulating Aux/IAA stability.^27^ Domains III and IV, also called PB1 domain, which share homology with the C-terminal domain (CTD) of ARF proteins. This domain mediates dimerization with ARFs and other Aux/IAAs.^28^ Variations in these domains contribute to functional divergence among Aux/IAA members. Aux/IAA proteins contain two nuclear localization signals (NLSs), located in domains II and IV, making them intrinsic nuclear proteins.^29^

## 3. Functional roles of non-canonical Aux/IAAs

Canonical Aux/IAA proteins, which possess all four conserved domains, have been extensively for their roles in mediating auxin response.^21,26^ However, there are also non-canonical Aux/IAAs that lack domain I or/and the typical conserved domain II. Increasing attention has been paid to uncovering the mechanism by which these non-canonical *Aux/IAAs* function in auxin signaling. Among the 29 Aux/IAA proteins in Arabidopsis, six are non-canonical, namely IAA20, IAA30, IAA31, IAA32, IAA33 and IAA34. Due to the complete absence of domain II, IAA20 is a long-lived protein and that does not degrade in response to auxin.^30^ Given their similar lack of domain II and belong to the same sub-family, it is reasonable to propose that the protein stability of IAA30 may be similar to that of IAA20. IAA31, which contains a region partially resembling domain II, also displays an extended half-life time, though auxin application promotes its degradation.^30^

**Figure 1.**
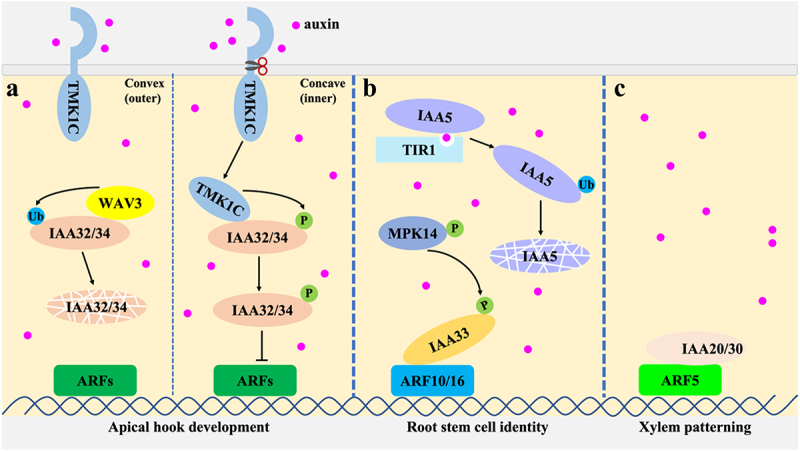
The non‐canonical auxin signaling pathways.

In contrast to the auxin-mediated rapid degradation of canonical Aux/IAA proteins, non-canonical Aux/IAAs such as IAA32/34 and IAA33 exhibit stabilized protein levels upon auxin treatment through distinct kinase-dependent phosphorylation processes.^31,32^ The differential auxin distribution between the concave (high auxin) and convex (low auxin) sides of the apical hook is critical for Arabidopsis hook formation. On the concave side, high auxin level inhibits cell elongation by suppressing ARF transcription factors through TMK1-medaited phosphorylation and stabilization of the auxin inhibitors IAA32 and IAA34.^31^ On the convex side, the WAV3 E3 ubiquitin ligase rather than the TIR1/AFBs is responsible for the degradation of IAA32 and IAA34, thereby releasing ARF activity to promote cell elongation.^33^ In the root, the non-canonical Aux/IAA protein IAA33 plays a critical role in maintaining distal stem cell identity under high auxin conditions. Auxin-induced accumulation of the IAA33 protein is mediated by the kinase MPK14, leading to the competition between IAA33 and canonical repressors IAA5 for binding to the transcriptional repressors ARF10 and ARF16. This competition effectively dampens auxin signaling, highlighting a unique regulatory mechanism where non-canonical IAAs modulate canonical pathways.^32^ Similarly, non-canonical IAA20 and IAA30 are essential for proper xylem patterning through an appropriate auxin response. Here, ARF5 activation upregulates IAA*20* and *IAA30* expression, and the accumulated proteins inhibit the activity of ARF5 through negative feedback regulation.^34^ This dynamic fine-tuning suggests that non-canonical *Aux/IAAs* act as critical rheostats in high-auxin contexts, such as the concave side of bending organs, root stem cell niches, and developing xylem axis. Together, these findings underscore the importance of non-canonical Aux/IAA proteins in maintaining cellular identity and tissue patterning under elevated auxin levels, revealing a layer of complexity in auxin signaling beyond canonical repression mechanisms (Figure 1).

(A) The TMK1-IAA32/34 module regulates apical hook development. On the convex side (low auxin), IAA32/34 are ubiquitinated by WAV3 E3 ligases and degraded, promoting growth. On the concave side (high auxin), auxin activates TMK1 receptor, leading to cleavage of its intracellular kinase domain (TMK1C). TMK1C phosphorylates IAA32/34, stabilizing them and inhibiting ARF-mediated transcription to suppress growth; (B) The MPK14-IAA33 module controls root stem cell identity. At high auxin levels, MPK14 phosphorylated and stabilized IAA33, which competes with IAA5 for binding to ARF10/16. Meanwhile, auxin promotes TIR1-mediated ubiquitination and degradation of IAA5, shifting the balance to IAA33-ARF10/16 interactions to maintain stem cell identity; (C) The IAA20/30-ARF5 module mediates xylem patterning via negative feedback. ARF5/MP induces expression of *IAA20/30*, which in turn inhibit ARF5/MP transcriptional activity, fine-tuning xylem differentiation.

## 4. Loss-of-function mutants: revealing redundancy

Extensive studies have demonstrated that single, double, and even high-order mutants of *Aux/IAA* genes often display no discernible phenotypical alterations or only mild defects compared to wild-type plants,^22,35–37^ highlighting the functional redundancy within this gene family. For example, the *iaa7*/*axr2–5* single mutant is nearly indistinguishable from wild type through development.^35^ Similarly, sub-clade double (*iaa8-1iaa9–1*) and triple (*iaa5-1iaa6-1iaa19–1*) mutants exhibit wild-type phenotypes, consistent with the absence of significant changes in their expression profile,^22^ further supporting functional overlap and compensation among *Aux/IAA* members in growth regulation. Higher-order mutants further illustrate this redundancy with nuanced phenotypic effects. A CRISPR-generated quadruple mutant (*iaa1iaa2iaa3iaa4*) shows only minor defects, while *iaa2iaa7iaa16* exhibits a pronounced shade avoidance-like phenotype. The sextuple mutant (*iaa1iaa2iaa3iaa4iaa7iaa16*) displays an even stronger shade avoidance response. Notably, the expression levels of *IAA2, IAA7 and IAA16* genes exhibited a higher fold increase in response to shade avoidance compared to other *Aux/IAA* members.^37^ Collectively, these findings demonstrate that while *Aux/IAA* genes exhibit intensive redundancy, individual members may assume more critical roles in specific contexts, likely dictated by their spatiotemporal expression patterns. Generating higher-order and diverse combinations of *aux/iaa* mutants will significantly advance our understanding of *Aux/IAA* functions and provides deeper insights into auxin signaling.

Despite their redundancy in growth regulation, some *Aux/IAA* loss-of-function mutants exhibit striking phenotypes under suboptimal conditions. For example, *IAA11* is critical for UV-AB acclimation and tolerance, as the *iaa11* mutant displays heightened susceptibility and cell death upon UV-AB stress.^38^ Similarly, *IAA15* regulates drought stress responses by inhibiting lateral root formation.^39^ The triple mutant *iaa5iaa6iaa19* is more sensitive to drought than wild-type plants, and this phenotype correlates with drought-induced expression of *IAA5* and *IAA19*, mediated by direct binding of transcription factors to their promoters.^40^ These findings highlight that *Aux/IAA* members can assume non-redundant, stress-specific roles, expanding their functional significance beyond developmental redundancy.

## 5. Gain-of-function mutants: deciphering specificity

While loss-of-function phenotypes for individual *Aux/IAA* genes remain scarce, gain-of-function mutants provide a valuable alternative for clarifying their specific roles. Among the 23 canonical *Aux/IAAs*, 10 possess one or more gain-of-function mutants (Table 1). These mutants typically carry single amino acid substitutions in the degron motif of conserved domain II, impairing their interaction with TIR1/AFB auxin receptors.^23,41,50^ As a result, the mutant proteins escape recognition and degradation by the 26S proteasome, leading to their abnormal accumulation and pleiotropic development defects. Some gain-of-function mutants share similar phenotypes, such as agravitropic roots and hypocotyls (*axr2–1*, *slr-1, axr5–1*),^41,44,46^ and photomorphogenesis phenotypes of dark-grown seedlings (*iaa3/shy2*, *iaa7/axr2*, *iaa17/axr3*),^35,52^ suggesting overlapping roles in certain *Aux/IAAs* members. However,divergent phenotypes also highlight gene-specific function, such as opposite effects on apical dominance (*iaa17/axr3* increased vs. *iaa28* reduced),^47,51^ hypocotyl elongation (*iaa18* longer vs. others shorter)^35,36,49^ and lateral root and root hair development (*iaa3/shy2*, *iaa7/axr2*, *iaa17/axr3* show distinct patterns).^35^ Collectively, the functional redundancy and specificity of *Aux/IAAs* ensure that auxin signaling maintains both stability and plasticity, enabling robust yet adaptable responses to developmental and environmental cues.

**Table 1.** Gain-of-function *Aux/IAA* mutants and their phenotypes.

Gene	Mutant(s)	Domain II Mutation	Phenotype	Reference
*IAA1/AXR5* GWPPVR	*axr5–1*	GWPPSR	Small rosette leaves, short petioles and inflorescence, reduced seed set, agravitropic and aphototropic root and hypocotyl	^41^
*IAA3/SHY2* GWPPVR	*shy2–1*	GWSPVR	Large cotyledons, short hypocotyls, dwarfism, curled-up leaves, reduced lateral and adventitious roots	^36,42^
	*shy2–2*	GWSPVR		
	*shy2–3*	EWPPVR		
	*shy2–6*	GWPLVR		
*IAA6/SHY1* GWPPVC	*shy1–1*	GWPPVR	Short hypocotyls, curled-up leaves	^42^
*IAA7/AXR2* GWPPVR	*axr2–1*	GWSPVR	Short hypocotyls, dwarfism, wavy leaves, increased lateral roots, no root hairs and adventitious roots, agravitropic roots and hypocotyls	^35,43,44^
*IAA12/BDL* GWPPIG	*bdl*	GWSPIG	No embryonic root, curled-up leaves	^45^
*IAA14/SLR* GWPPVR	*slr-1*	GWPSVR	No lateral roots, reduced root hairs, agravitropic roots and hypocotyls	^46^
*IAA17/AXR3* GWPPVR	*axr3–1*	GWPLVR	Increased apical dominance, curled-up leaves, short hypocotyls, more adventitious roots, no root hairs, agravitropic root	^47,48^
	*axr3–3*	GWPPGR		
*IAA18* GWPPVR	*iaa18–1*	EWPPVR	Long hypocotyls, fused cotyledons, short roots, curled-up leaves	^49^
*IAA19/MSG2* GWPPVC	*msg2–1*	GWPSVC	Agravitropic and aphototropic hypocotyl, reduced lateral roots	^50^
	*msg2–2*	RWPPVC		
	*msg2–3*	GWPLVC		
	*msg2–4*	GWLPVC		
*IAA28/IAR2* GWPPVR	*iaa28–1*	GWLPVR	Reduced lateral roots, decreased shoot apical dominance	^51^

## 6. Expression patterning of Aux/IAAs

Transcriptomic analyses reveal that auxin-responsive genes in Arabidopsis roots exhibit strong tissue-specific expression pattern^53^，underscoring the importance of spatial regulation in auxin signaling. The expression pattern of *Aux/IAA* genes may play a crucial role in determining their function specificity. Their spatial and temporal expression profiles often correlate well with their physiological roles. For instance, certain *Aux/IAA* genes *(e.g. IAA1, IAA3, IAA6, IAA7*) are expressed throughout plant development,^20^ consistent with their pleiotropic mutant phenotypes. Some exhibit more restricted expression patterns, such as *IAA32* and *IAA34* in the apical hook,^31^ *IAA20* and *IAA30* in the root xylem axis^34^ and *IAA33* in root.^32^ Promoter-swap experiments further highlight the importance of expression pattern in Aux/IAA function. For example, although the *iaa3/shy2–2* mutant does not exhibit embryonic defects, the expression of a stabilized shy2–2 repressor under the *IAA12*/*BDL* promoter results in a rootless phenotype and postembryonic growth abnormalities resembling *bdl* phenotype. Conversely, when a stabilized bdl protein is driven by the *SHY2* promoter, the transgenic plants exhibit short hypocotyls and shoots that are not observed in *bdl* phenotypes.^54^ Similarly, depending on the promoter used, tissue-specific expression of stabilized *iaa14/slr* can either block lateral root initiation or disrupt lateral root primordium development.^55^ Additionally, the expression of stabilized *aux/iaa*, *axr2–1*, *slr-1* or *msg2–1* under the *MSG2* promoter recapitulates the *msg2–1* phenotype. These findings collectively demonstrate that promoter-driven expression patterns are key determinants of *Aux/IAA* mutant phenotypes.^56^ However, proteins properties, interactions between specific Aux/IAAs and TIR1/AFBs, and their combinatorial effects with ARFs also contribute to functional specificity.^57^ Thus, the diversity of *Aux/IAA* functions arises from a combination of expression patterns, protein stability, and interaction networks.

## 7. Conclusion and future perspective

The Aux/IAA gene family plays a central role in auxin signaling, exhibiting both functional redundancy and specificity to precisely regulate plant growth and development. Canonical Aux/IAA proteins, with four conserved domains, modulate auxin responses primarily through the well-characterized TIR1/AFB-Aux/IAA-ARF pathway. In contrast, non-canonical Aux/IAAs, which lack key domains, influence auxin signaling via alternative mechanisms, such as kinase-mediated stabilization and competition with canonical repressors. Genetic studies reveal that loss-of-function mutants display extensive redundancy among Aux/IAA members, whereas gain-of-function mutants uncover gene-specific roles. Additionally, the spatiotemporal expression patterns of *Aux/IAA* genes likely contribute to their functional diversification.

However, several key questions remain to be answered? How do non-canonical *Aux/IAAs* functionally interact with canonical *Aux/IAAs* in auxin signaling? Do non-canonical Aux/IAAs influence the AC activity of TIR1/AFBs? Are there additional kinases or E3 ligases involved in regulating Aux/IAA protein stability? What molecular mechanisms determine the degree of functional redundancy and specificity among *Aux/IAA* members? Is it possible to generate higher-order mutants by knocking out all *Aux/IAA* genes? If so, how would this affect auxin signaling? Why do certain *Aux/IAAs* exhibit specialized roles under sub-optimal conditions?

The *Aux/IAA* family exemplifies how plants enable both robustness and flexibility in auxin signaling. Future studies integrating genetics, biochemistry, and systems biology will deepen our understanding of auxin-mediated growth regulation and open new avenues for manipulating plant development and stress adaptation.
